# Evolution of burnout syndrome in Spanish healthcare professionals during and after the COVID-19 pandemic: psychosocial variables involved

**DOI:** 10.3389/fmed.2025.1522134

**Published:** 2025-02-07

**Authors:** Fernanda Gil-Almagro, F. Javier Carmona-Monge, Fernando J. García-Hedrera, Cecilia Peñacoba-Puente

**Affiliations:** ^1^Intensive Care Unit, Hospital Universitario Fundación Alcorcón, Madrid, Spain; ^2^Department of Nursing, Universidad Francisco de Vitoria, Madrid, Spain; ^3^Psychology Department, Faculty of Health Sciences, Universidad Rey Juan Carlos, Madrid, Spain; ^4^Department of Anaesthesia, Hospital Universitario Santiago de Compostela, Santiago de Compostela, Spain

**Keywords:** burnout, healthcare worker, COVID-19, post-pandemic, emotional exhaustion, depersonalization, self-fulfillment

## Abstract

**Introduction:**

Evidence shows that throughout the COVID-19 pandemic, healthcare workers have experienced high levels of burnout. The preceding literature also points to the need to consider the three elements of burnout independently, as they appear to have different evolutionary trends and possibly different buffering and amplifying variables, although these aspects have hardly been explored.

**Methods:**

The aim of the present investigation is precisely to shed light on these latter issues. It is a prospective study, carried out in 256 healthcare workers in Spain during three time points in relation to the COVID-19 pandemic: (1) (T1) between 5 May and 21 June 2020 (final phase of the state of alarm declared in Spain on 14 March), (2) (T2) 6 months after the end of the state of alarm (January–April 2021), and (3) (T3) 1 year after this second evaluation (April–July 2022). The different components of burnout syndrome (emotional exhaustion, depersonalization and self-fulfillment) were assessed at the second and third time points. Together with sociodemographic and occupational data (age, gender, professional category, years of experience, hours of work), anxiety, depression, stress, family support, friends’ support, and self-efficacy were assessed at the first time point. At the second time point, cognitive fusion and resilience were assessed. At the third time point, optimism and hopelessness were assessed.

**Results:**

The results show significant decreases in burnout syndrome (*p* < 0.001). However, when observing the evolution of each of the dimensions, it can be seen that emotional exhaustion has significantly decreased (*p* < 0.001), while an increase in depersonalization (*p* < 0.001) and a decrease in self-fulfillment (all *p* < 0.001) are observed. The results of the repeated measures General Linear Models, after controlling for the effect of the covariates show that the evolution of emotional exhaustion is associated with the levels of depression at T1 (*p* = 0.031), of cognitive fusion at T2 (*p* < 0.001) and of resilience at T2 (*p* = 0.039). The evolution of depersonalization is associated with levels of hopelessness at T2 (*p* = 0.042). Finally, the evolution of self- fulfillment is associated with levels of optimism at T3 (*p* = 0.043) and hopelessness at T3 (*p* = 0.019).

**Discussion:**

The results highlight the need to attend to the three components of burnout in a differentiated manner. Our results indicate that, during the COVID-19 pandemic, although overall burnout levels tend to decrease, it is actually emotional exhaustion that decreases, but levels of depersonalization increase and self- fulfillment decreases. In addition, the data point to the different personality factors involved in each of the dimensions. While the evolution of emotional exhaustion seems to be more affected by the levels of symptomatology (i.e., depression) at the onset of the pandemic, and of the inability to handle intrusive thoughts (i.e., cognitive fusion), the evolution of depersonalization and self- fulfillment are more related to long-term cognitive-emotional personality variables such as optimism and hopelessness.

**Practical implications:**

The results found have important practical implications for the prevention of each of the implicated components of the syndrome. Although further research is needed, emotional exhaustion is shown to be one of the dimensions affected in the short term and intervention programs aimed at reducing anxiety and depression at times of acute stress (onset of the COVID-19 pandemic), including thought management, seem fundamental. Depersonalization and decreased self-fulfillment do not seem to respond to the same pattern. They are shown as results of a chronification of a poor management of emotional exhaustion, and in the case of their appearance, given the variables associated with their evolution (i.e., optimism and hopelessness), therapies more focused on the meaning of existence, such as Acceptance and Commitment Therapy, could be useful.

## 1 Introduction

The COVID-19 pandemic had a profound and lasting impact on healthcare workers (HCW) worldwide (1, 2). During this period there was an unprecedented surge of critically ill patients, and many HCW worked in high-risk environments, most of the time with limited resources, personal protective equipment (PPE), and overwhelming workloads. The emotional and physical toll was immense, leading to increased stress, burnout, and mental health challenges (insomnia, depression, anxiety) (3–6). HCW faced exposure to the virus, risking their own health and that of their families. The pandemic also highlighted systemic issues in healthcare systems, such as staffing shortages and underfunding, further straining workers (7). Despite these challenges, HCW showed remarkable resilience, adapting to new protocols and technologies to provide care in an evolving crisis.

Burnout syndrome has been a significant issue among HCW for decades, characterized by chronic workplace stress that has not been successfully managed. Before the COVID-19 pandemic, burnout was already widespread due to a combination of different factors such as heavy workloads, long hours, insufficient staffing, administrative burdens, and the emotional toll of caring for patients in high-pressure environments. HCW often experienced emotional exhaustion, feeling drained and unable to emotionally engage with their work. This was accompanied by depersonalization, being detached or even cynical about the patients they were treating. Additionally, a diminished sense of personal accomplishment was common (8–10).

However, the COVID-19 pandemic drastically exacerbated these pre-existing issues. Emotional exhaustion was increased as HCW were working in an unprecedented crisis, dealing with an overwhelming influx of critically ill patients. The continuous exposure to death and suffering, combined with fears of contracting the virus or spreading it to loved ones, further intensified their stress levels. HCW faced constant moral dilemmas, such as choosing which patients received ventilators due to shortages, leading to feelings of guilt and helplessness (11, 12). The post-pandemic situation made evident the systemic flaws in healthcare systems worldwide, such as chronic underfunding, inadequate staffing, and lack of mental health support.

During the COVID-19 pandemic, the concepts of resilience and burnout became central to the experience of HCW. Resilience refers to the ability to adapt and recover from adversity, and many HCW demonstrated remarkable resilience despite the extreme pressures they faced. In terms of resilience, research highlights its critical role as a buffer against burnout. Workers who demonstrated higher resilience were better equipped to cope with pandemic-related stress. Personal resilience can be nurtured through various coping mechanisms, such as problem-focused and emotion-focused strategies, social support, physical self-care, and distancing from work. Organizational resilience also played a key role, with studies emphasizing the importance of leadership support, team cohesion, and access to mental health resources in fostering resilience among HCW. Individuals who were able to maintain their social connections and prioritize self-care were found to have lower rates of burnout and emotional exhaustion (13, 14).

However, the prolonged nature of the pandemic also led to an increase in hopelessness arising from a sense of helplessness in the face of relentless suffering and loss, coupled with the inability to see an end to the crisis. Research indicates that workers experiencing high levels of burnout were more likely to feel hopeless, as they became emotionally detached from their work and doubted their ability to make a meaningful impact. This hopelessness was closely linked to feelings of moral distress, especially when HCW were forced to make difficult decisions about patient care due to resource constraints (15–18).

Despite these challenges, some studies offer a hopeful outlook, suggesting that workplace interventions aimed at improving psychological safety, promoting work-life balance, and providing emotional support can reduce burnout and prevent hopelessness. Addressing systemic issues, such as staffing shortages and access to mental health care, is essential for long-term resilience and recovery in post-pandemic healthcare environments. Moving forward, the research emphasizes the need for both individual coping strategies and structural reforms to support HCW in rebuilding resilience and overcoming burnout (19–22).

The aim of this research is to analyze the evolution of burnout syndrome in health professionals in Spain from the beginning of the COVID-19 pandemic until a year and a half later (the post-pandemic stage). The analysis of the evolution will be carried out by looking, in a differential way, at the three components involved (emotional exhaustion, depersonalization and self-fulfillment). On the other hand, the psychosocial variables, including personality and emotional symptomatology, involved in the evolution of each of the components of burnout will be analyzed.

In particular, and taking into account previous literature, the following hypotheses are put forward: (1) A decrease in burnout syndrome is expected over the time considered in the present research (from 6 months after the beginning of the COVID-19 pandemic to a year and a half later), (2) A different evolution is expected with respect to the three components of burnout with the most significant decreases occurring in the emotional exhaustion component, as it is hypothesized, at the same time, the component initially most affected by the COVID-19 pandemic, (3) Anxiety, depression, stress, cognitive fusion and hopelessness are expected to be risk variables. Thus, HCW with high scores on the above variables will show higher values of burnout (and its components) at each of the time points considered and will show a worse evolution, (4) Social support, resilience, self-efficacy and optimism will be shown to be protective variables. HCW with high scores on the above variables will show lower burnout scores (and in its components) at the time points considered, as well as a better evolution of the syndrome, (5) A differential role of the risk and protective variables considered on the different components of burnout syndrome is expected, hypothesizing a greater effect on emotional exhaustion with respect to the rest of the dimensions.

## 2 Methodology

### 2.1 Design

A prospective longitudinal study was carried out, with data collected at three distinct time points: (1) from May 1st to June 21st, 2020 (during the final phase of the state of alarm declared in Spain on March 14th), (2) 6 months after the state of alarm ended (from January to April 2021), and (3) 1 year after the second evaluation (from April to July 2022). During the first data collection period, Spain was under a state of alarm, which included a lockdown lasting until June 21st, 2020.

Burnout was assessed in participants during the second and third time points. Furthermore, various sociodemographic, occupational, and psychosocial variables were measured at all three time points (see the Instruments section for more details). Table 1 shows the evaluation periods and variables included in the study.

**Table 1:** Socio-demographic, occupational, and psychosocial variables collected at the three points in time of the study.

	Variables collected at different time point		
	1st evaluation period	2nd evaluation period	3rd evaluation period
	May 5th–June 21st (2020)	January 9th–April 9th (2021)	April 11th–July 15th (2022)
Symptoms	Anxiety, depression, stress	Burnout	Burnout
Sociodemographics	Age, gender, years of experience, working horus, professional category, service.		
Personality	Resilience Self-efficacy Social Support	Cognitive fusion	Optimism Hopelessness

### 2.2 Procedure and participants

Data were gathered using a custom-designed online electronic questionnaire. Participants were asked to provide informed consent and their email addresses if they wished to participate in future phases of the study.

The sample consisted of HCW from the Spanish National Health System. A probabilistic convenience sampling method was employed with the following inclusion criteria: being a nurse, physician, or nursing care technician; working in a public or private healthcare service of the Spanish National Health System; being 18 years or older; and having direct contact with COVID-19 patients. Exclusion criteria included being on sick leave during the data collection period or working in healthcare management.

A minimum study population of 120 was required, based on reference figures for prospective studies. Considering the challenging context of the COVID-19 pandemic and the longitudinal design of the study, a larger initial sample size of 400 participants was targeted. In the first data collection phase, 1.374 HCWs were included. Of these, 881 participated in the second phase, and 259 continued through to the third phase, resulting in a final sample size significantly exceeding the initially estimated 120 participants.

To recruit participants, the questionnaire link was sent to HCW in the Spanish health system, both public and private, through social media platforms (Facebook, LinkedIn, Twitter, WhatsApp), and corporate emails from various public and private healthcare services. For the second and third phases, emails from participants in the first phase were used to invite them to continue their participation.

### 2.3 Ethical considerations

The study was approved by the Hospital Ethics and Research Committee. In addition, the present study has received the scientific endorsement of the Spanish Society of Intensive and Coronary Care Nursing (SEEIUC). Participants were informed of the aim of the study prior to providing their written informed consent to take part. They were also informed that they may withdraw from the study at any point. They were also informed that their responses would be kept completely anonymous and used only for research purposes.

### 2.4 Variables and instruments

#### 2.4.1 Sociodemographic and occupational variables [time point 1]

The research team created a customized questionnaire to gather this information. It included sociodemographic details such as age, gender, as well as work-related data like job category, department and years of professional experience.

*Burnout [time point 2 and time point 3]:* the Maslach Burnout Inventory-Human Services Survey (MBI-HSS) (23) was used in its Spanish version (24). This 22-item scale adopts a 7-point Likert-type response format ranging from 0 (never) to 6 (every day). The instrument assesses three dimensions or subscales of burnout (i.e., emotional exhaustion, depersonalization, and decreased self-fulfillment). In our study, Cronbach’s *α* for the instrument used was 0.88 for the full scale. In relation to the subscales, Cronbach’s α was 0.90 for emotional exhaustion, 0.72 for depersonalization and 0.84 for decreased self-fulfillment. According to the literature, the Maslach Bournout Inventory instrument defines a high emotional exhaustion for scores above 27, a high level of work depersonalization with scores above 10 and a low personal fulfillment with scores between 0 and 33, scores above 40 are considered to be a sign of high personal fulfillment (24–26).

*Anxiety, depression and stress [time point 1]:* the depression, anxiety and stress scale (DASS-21) (27) was used in its Spanish version (28), a scale designed to evaluate states of depression, anxiety and stress. Each dimension consists of seven items with a Likert-type response format of four alternatives from 0 (“it has not happened to me”) to 3 (“it has happened to me a lot” or “most of the time”). The score for each of the dimensions ranges from 0 to 21 points. Cronbach’s alpha in our study is 0.82 for stress, 0.77 for anxiety and 0.80 for depression.

*Social support [time point 1]:* measured using the Spanish version (29) of the Multidimensional Scale of Perceived Social Support (MSPSS) (30), which is composed by 12 items divided into three dimensions: family, friends and significant others, with a 7-point Likert-type response scale (from 1 “completely disagree” to 7 “completely agree”). The final score comes from the sum of its three subscales. The instrument has good properties (31, 32), for our study its reliability was *α* = 0.85 for the general questionnaire, while for the subscales the α values obtained were 0.81, for family, 0.82 for friends and 0.79 for significant others.

*Self-efficacy [time point 1]:* the General Self-Efficacy Scale (GSES) (33) was used in its Spanish version (34), composed of 10 items that measure the perception of competence to handle life situations, with a 4-point Likert-type response format between 1 (“not at all true”) and 4 (“completely true”). The total score ranges from 10 to 40; a higher score indicates better levels of self-efficacy. Cronbach’s alpha was 0.86 in our study.

*Resilience [time point 1]:* the Spanish adaptation of the Resilience Scale (RS-14) (35) was used, consisting of 14 Likert-type items with 7 response options, ranging from 1 (“strongly disagree”) to 7 (“strongly agree”). The total possible score ranges from 14 to 98, with higher scores reflecting greater levels of resilience. In our study, the reliability coefficient (*α*) was 0.94.

*Cognitive fusion [time point 2]:* the Spanish version (36) of the Cognitive Fusion Questionnaire (CFQ) (37) was administered. It is made up of 7 Likert-type items with 7 response options, ranging from 1 “never” to 7 “always,” that higher scale scores imply a higher degree of cognitive fusion. A Cronbach’s *α* of 0.97 was obtained for our study.

*Optimism [time point 3]:* the Spanish version (38) of The Life Orientation Test-Revised (LOT-R) (39) was used to measure dispositional optimism. It is a 10-item on a 5-point scale from 0 (strongly disagree) to 4 (strongly agree) that assesses dispositional optimism through a single factor. Total score ranges from 0 to 24; higher scores indicate greater optimism. Previous studies have shown good psychometric qualities for this instrument (38, 39). The Cronbach’s Alpha value in our sample is 0.79.

*Hopelessness [time point 3]:* the hopelessness questionnaire of Beck was used (40) in its Spanish version (41). The scale is designed to measure the cognitive, affective, and motivational dimensions of hopelessness during the last 7 days. The scale is a 20-item, self-administered questionnaire. All items are scored on a true–false rating scale. After recoding negatively worded items, the number of endorsed items is combined to a sum-score; the higher scores the greater hopelessness levels. The participants were classified into: no hopelessness (scores 0–3), mild hopelessness (scores 4–8), moderate hopelessness (scores 9–14) and severe hopelessness (scores 15–20) (40). Cronbach’s *α* in this sample was 0.87.

### 2.5 Data analysis

In the descriptive analysis of the sample, summary statistics were applied as appropriate. The categorical variables were described by means of absolute frequencies (n) and relative (%) ones. Continuous variables were described using the using the mean and standard deviation. A Chi-square independence test was used to examine the association between categorical variables. *T*-test and one-way ANOVA were used to compare the distribution between 2 or more than 2 groups of continuous variables. Pearson’s correlation was used to analyse the relationship between two continuous variables. To investigate changes in burnout and its dimensions, repeated measures ANOVAs were used. To examine whether the burnout evolution depends on psychosocial variables, different analyses using General Linear Models were employed using as an intrasubject variable the dimensions of burnout syndrome at each time point and as an intersubject factor the psychosocial and emotional symptomatology variables considered. For the establishment of high and low values for each of the psychosocial variables (dividing continuous variables into two categories), a statistical criterion was used. Specifically, the median of the variable’s distribution was used as the cut-off point. All analyses were conducted using SPSS version 28 (IBM Corp, 2021).

## 3 Results

### 3.1 Description of the sociodemographic and occupational variables of the sample

Table 2 presents the sociodemographic, occupational, and health data for the 259 participating HCW. As shown in Table 2, most HCW were women (81.5%) and nurses (59.1%). The Critical Care Unit (CCU) was the most common service area (37%), followed by hospitalization (28.2%). The participants had about 10 years of work experience. A significant portion of the sample (75.7%) expressed high concern about contracting COVID-19 themselves or a family member. Additionally, approximately 20% sought psychological support.

**Table 2:** Socio-demographic and occupational characteristics of participants.

		*f* (%)	Mean (SD)
Age			43.68 (9.78)
Experience (years)			11.66 (9.20)
Gender	Man	48 (18.5%)	
	Woman	211 (81.5%)	
Professional category	Physician	65 (25.1%)	
	Nurse	153 (59.1%)	
	Nursing tec^1^	41 (15.8%)	
Speciality	ICU	96 (37.1%)	
	Hospitalization	73 (28.2%)	
	Emergencies	38 (14.7%)	
	Primary care	42 (16.2%)	
	Others	10 (3.8%)	

^¹Nursing Technician.^

### 3.2 Evolution of burnout syndrome and each of its dimensions

As can be seen in Table 3, the results show significant decreases in burnout syndrome (*p* < 0.001). Assessing the evolution of each dimension of burnout, as shown in Table 3, emotional exhaustion has decreased significantly, while an increase in depersonalization (*p* < 0.001) and a decrease in self-fulfillment (all *p* < 0.001) can be observed.

**Table 3:** Changes in burnout dimensions (emotional exhaustion, depersonalization, self-fulfillment) across three time points.

	T2	T3	95% CI for the difference of means	*t*	*p*
	Mean (SD)	Mean (SD)			
Emotional exhaustion	26.81 (13.62)	22.28 (14.18)	[2.96/6.09]	5.689	<0.001
Depersonalization	7.32 (6.57)	9.16 (6.99)	[−2.63/−1.04]	−4.549	<0.001
Self-fulfillment	35 (8.67)	30.55 (9.99)	[3.16/5.73]	6.813	<0.001
Burnout (total)	69.13 (18.37)	62 (18.80)	[4.90/9.36]	6.301	<0.001

### 3.3 Relationships between socio-demographic and occupational variables and burnout dimensions

**Figure 1:**
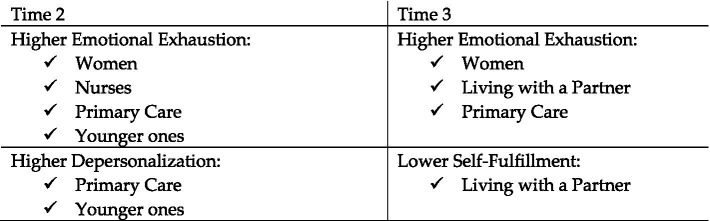
Summary of the risk profiles (socio-demographic and occupational variables) for the different dimensions of burnout at different time points.

Table 4 shows statistical results of the relationship between socio-demographic and occupational variables and the three dimensions of burnout at the second (Time 2) and third time (Time 3) points. The results show significant relationships between age, gender, cohabitation as a couple, occupational category and service. A graphical representation of these results is shown in Figure 1. As can be seen in Table 4 and Figure 1, the following results are worth noting:

**Table 4:** Relationships between socio-demographic and occupational variables and burnout dimensions over the time periods.

	T2			T3		
	Emotional exhaustion	Depersonalization	Self-fulfillment	Emotional exhaustion	Depersonalization	Self-fulfillment
	R^2^/Mean (SD)	R^2^/Mean (SD)	R^2^/Mean (SD)	R^2^/Mean (SD)	R^2^/Mean (SD)	R^2^/Mean (SD)
Age	R^2^ = −0.186 *p* = 0.003	R^2^ = −0.175 *p* = 0.005	R^2^ = 0.067 *p* = 0.281	R^2^ = −0.081 *p* = 0.195	R^2^ = −0.102 *p* = 0.101	R^2^ = 0.045 *p* = 0.466
Gender						
Men (*n* = 48)	20.93 (13.98)	7.39 (7.02)	36.20 (9.80)	18.58 (15.39)	8.77 (7.13)	33.08 (9.73)
Women (*n* = 211)	28.15 (13.21)	7.30 (6.48)	34.72 (8.40)	23.12 (13.80)	9.25 (6.97)	29.97 (9.98)
	*p* = 0.001	*p* = 0.930	*p* = 0.286	*p* = 0.045	*p* = 0.668	*p* = 0.052
Cohabitation as a couple						
without a partner (*n* = 77)	26.97 (13.72)	7.54 (7.17)	35.02 (9.17)	19.37 (13.38)	8.40 (6.93)	32.46 (9.67)
with a partner (*n* = 182)	26.74 (13.62)	7.22 (6.32)	34.98 (8.48)	23.51 (14.37)	9.48 (7.01)	29.74 (10.04)
	*p* = 0.903	*p* = 0.721	*p* = 0.975	*p* = 0.032	*p* = 0.256	*p* = 0.045
Professional category						
Physician (*n* = 65) (1)	27.20 (13.60)	7.53 (6.56)	34 (9.92)	22.01 (14.63)	8.64 (6.78)	32.24 (9.18)
Nurse (*n* = 151) (2)	28.19 (13.40)	7.57 (6.77)	35.25 (7.80)	23.25 (13.78)	9.65 (7.03)	29.65 (9.97)
Technician (*n* = 43) (3)	21.39 (13.42)	6.09 (5.85)	35.62 (9.65)	19.20 (14.81)	8.20 (7.16)	31.13 (11.06)
	*p* = 0.015 (2, 3)	*p* = 0.409	*p* = 0.547	*p* = 0.270	*p* = 0.388	*p* = 0.199
Service						
CCU (*n* = 93) (1)	27.69 (13.19)	6.09 (5.94)	34.89 (8.28)	22.20 (14.92)	8.26 (7.02)	30.52 (10.79)
Hospitalization (*n* = 87) (2)	23.20 (13.88)	6.89 (6.76)	34.58 (10.31)	19.82 (13.01)	9.24 (7)	30.78 (9.20)
Emergencies (*n* = 34) (3)	25.41 (13.77)	8.61 (6.48)	36.02 (7.56)	22.50 (13.77)	9.79 (6.41)	27.94 (10.13)
Primary care (*n* = 45) (4)	32.88 (11.83)	9.86 (6.92)	35.20 (6.84)	27.29 (14.43)	10.52 (7.32)	32.13 (9.69)
	*p* = 0.001 (2, 4)	*p* = 0.009 (1, 4)	*p* = 0.872	*p* = 0.043 (2, 4)	*p* = 0.327	*p* = 0.328
Professional experience	R^2^ = −0.025 *p* = 0.684	R^2^ = −0.111 *p* = 0.073	R^2^ = 0.062 *p* = 0.322	R^2^ = −0.032 *p* = 0.603	R^2^ = −0.044 *p* = 0.477	R^2^ = 0.013 *p* = 0.832

Negative relationships are observed between age and both emotional exhaustion and depersonalization at Time 2. Women experience higher levels of emotional exhaustion at both Time 2 (Cohen’s d = 0.53) and Time 3 (Cohen’s d = 0.31). Living with a partner is related to higher levels of emotional exhaustion (Cohen’s d = 0.29) and lower self-fulfillment scores (Cohen’s d = 0.27) at Time 3. Higher emotional exhaustion is observed in nurses compared to technical support staff (Cohen’s d = 0.51) at Time 2. Higher scores are observed in primary care compared to hospitalization in emotional exhaustion at both Time 2 (Cohen’s d = 0.75) and Time 3 (Cohen’s d = 0.54). Finally, higher depersonalization are observed in primary care than in CCU (Cohen’s d = 0.58) at Time 2.

The largest effect sizes (Cohen’s d), medium-high effect sizes, are observed at Time 2, especially with regard to the service in which the job is performed, professional category and gender.

### 3.4 Evolution of the three burnout dimensions including intersubject factor (anxiety, depression, stress, family support, support from friends, self-efficacy, cognitive fusion, resilience, optimism and hopelessness)

**Figure 2:**
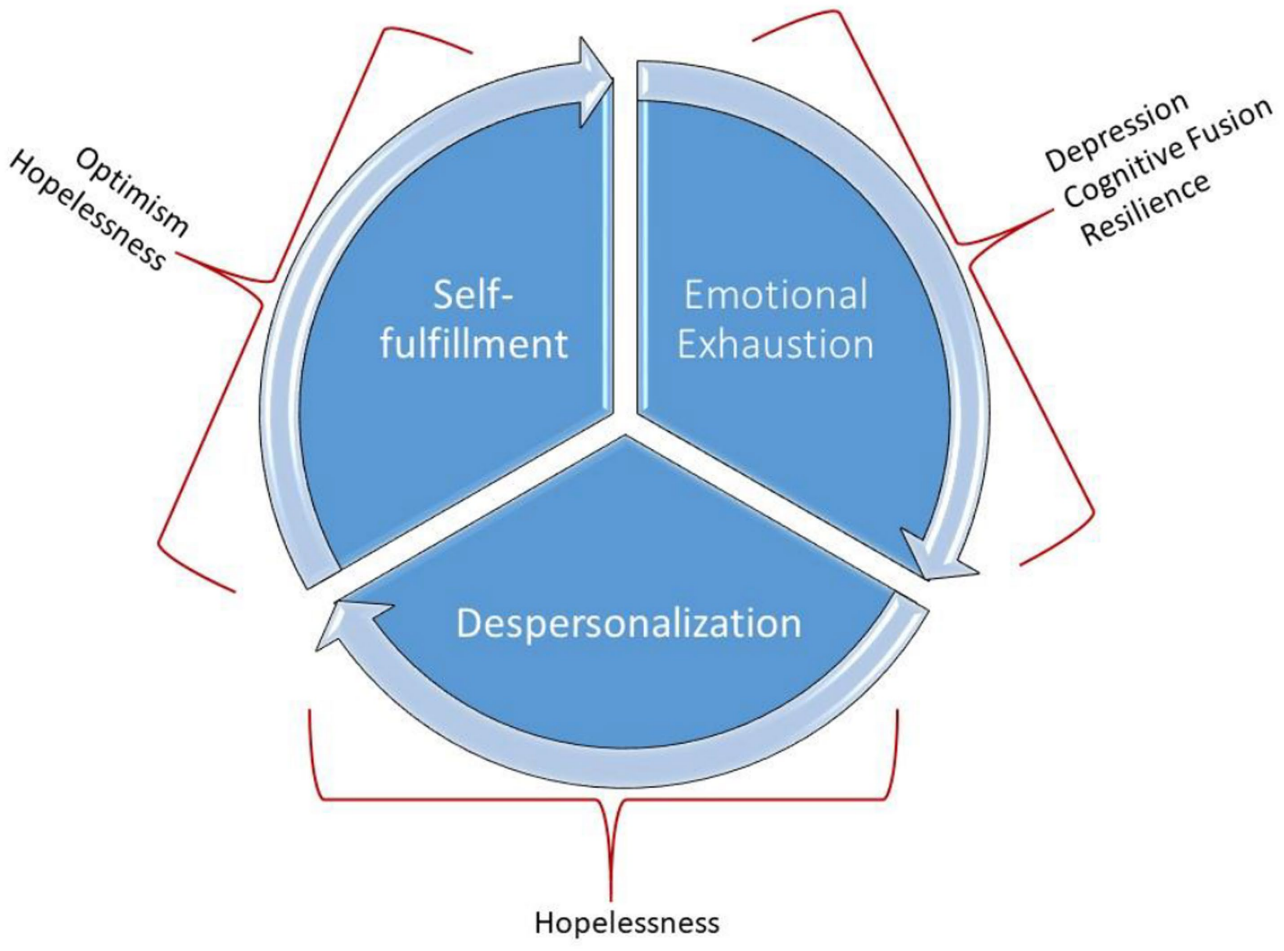
Graphical representation of the psychosocial variables that influence the different dimensions of burnout and its evolution over time.

Table 5 shows the statistically significant results in relation to the evolution of the three dimensions of burnout, including as an inter-subject factor, the different symptomatology variables considered (anxiety, depression, stress), as well as the psychosocial variables (family support, support from friends, self-efficacy, cognitive fusion, resilience, optimism, and hopelessness). For a better understanding, Figure 2 shows graphically the psychosocial variables related to each of the burnout dimensions and their evolution over time.

**Table 5:** Evolution of the three burnout dimensions over the time periods including psychosocial variables as intersubject factor (only significant interaction results are shown).

	T2 Mean (SD)	T3 Mean (SD)	P (T2/T3)				
Emotional exhaustion							
Depression (L)	21.06 (12.04)	18.11 (13.09)	0.001				
Depression (H)	33.57 (12.23)	27.19 (13.90)	<0.001				
	*P* < 0.001 *η*^2^ = 0.780	*P* < 0.001 *η*^2^ = 0.654					
Factor				*F* = 34.655	*P* < 0.001	*η*^2^ = 0.119	1-β = 1
Factor*Depression				*F* = 4.695	*P* = 0.031	*η*^2^ = 0.018	1-β = 0.579
Cognitive fusion (L)	19.41 (11.51)	17.92 (13.77)	0.166				
Cognitive fusion (H)	35.39 (10.52)	27.34 (12.97)	<0.001				
	*P* < 0.001 *η*^2^ = 0.827	*P* < 0.001 *η*^2^ = 0.660					
Factor				*F* = 38.061	*p* < 0.001	*η*^2^ = 0.129	1-β = 1
Factor*Cog. Fusion				*F* = 18.004	*p* < 0.001	*η*^2^ = 0.065	1-β = 0.988
Resilience (L)	29.79 (13.21)	23.83 (14.31)	<0.001				
Resilience (H)	22.84 (13.20)	20.21 (13.81)	0.025				
	*P* < 0.001 *η*^2^ = 0.564	*P* = 0.042 *η*^2^ = 0.470					
Factor				*F* = 28.840	*P* < 0.001	*η*^2^ = 0.101	1-β =1
Factor*Resilience				*F* = 4.320	P = 0.039	*η*^2^ = 0.017	1-β = 0.544
Depersonalization							
Hopelessness (L)	6.08 (5.76)	7.26 (6.28)	0.022				
Hopelessness (H)	9.25 (7.29)	12.12 (7.04)	<0.001				
	*p* < 0.001 *η*^2^ = 0.451	*p* < 0.001 *η*^2^ = 0.571					
Factor				*F* = 24.153	*p* < 0.001	*η*^2^ = 0.086	1-β = 0.998
Factor* Hopelessness				*F* = 4.185	*p* < 0.042	*η*^2^ = 0.016	1-β = 0.531
Self-fulfillment							
Optimism (L)	33.18 (8.65)	27.36 (9.59)	*P* < 0.001				
Optimism (H)	36.66 (8.39)	33.48 (9.48)	P = 0.001				
	*P* < 0.001 *η*^2^ = 0.761	*P* < 0.001 *η*^2^ = 0.596					
Factor				*F* = 48.076	*p* < 0.001	*η*^2^ = 0.158	1-β =1
Factor* Optimism				*F* = 4.121	P = 0.043	*η*^2^ = 0.016	1-β = 0.525
Hopelessness (L)	36.25 (8.73)	33.03 (9.57)	*P* < 0.001				
Hopelessness (H)	33.03 (8.26)	26.67 (9.44)	*P* < 0.001				
	*P* < 0.001 *η*^2^ = 0.854	*P* < 0.001 *η*^2^ = 0.755					
Factor				*F* = 52.221	*p* < 0.001	*η*^2^ = 0.169	1-β =1
Factor* Hopeless.				*F* = 5.61	P = 0.019	*η*^2^ = 0.021	1-β = 0.656

L, Low levels; H, High levels.

As shown in Table 5, significant interactions between depression, cognitive fusion and resilience for the evolution of emotional exhaustion, hopelessness for the evolution of depersonalization, and optimism and hopelessness for the evolution of self-fulfillment were found. In all cases, low effect sizes are observed. In other words, the consideration of the different inter-subject factors, although statistically significant, does not contribute much to the variability in the evolution over time of the three dimensions of burnout.

Additionally, partial effects of the intersubject variables considered on burnout symptomatology are observed in each of the time points considered. The variables involved for each dimension of burnout are the same as those observed in the case of their evolution, showing a larger effect size.

As shown in Table 5, with respect to emotional exhaustion, in both Time 2 and Time 3, higher scores are observed in participants with greater cognitive fusion, greater depression and less resilience. Effect sizes are larger at Time 2.

With regard to depersonalization, as shown in Table 5, higher scores are observed in participants with higher hopelessness at both Time 2 and Time 3, in this case the effect size being higher at Time 3. Finally, with regard to self-fulfillment, Table 5 shown lower scores in participants with lower optimism and higher hopelessness, both at Time 2 and Time 3. In the case of self-fulfillment, effect sizes are larger at Time 2.

## 4 Discussion

The results of our study underscore the importance of differentially addressing the three components of burnout: emotional exhaustion, depersonalization, and self-fulfillment. The results allow us to test the hypotheses put forward in this respect. Indeed, as we hypothesized in hypothesis 1, a decrease in burnout is observed, and a differential evolution in each of the components of the syndrome is also found (hypothesis 2). The results found corroborate the hypotheses put forward, providing interesting nuances that should be explored in greater depth. Thus, one of the most novel aspects with respect to the evolution of the dimensions is the fact that while emotional exhaustion (indeed, the most affected component of burnout as hypothesized) decreases, in coherence with the general decrease in burnout syndrome, depersonalization increases and personal fulfillment decreases. On the other hand, depersonalization is affected to a similar extent to emotional exhaustion, although in opposite directions (while the former decreases, the latter increases).

The results found can be supported by a number of different studies who report that during the COVID-19 pandemic, health care professionals suffered an increase in burnout over the previously recorded prevalence, mainly due to increased emotional exhaustion (42), however, these levels seem to decrease in the studies recorded after the passage of the pandemic due again to a decrease in emotional exhaustion. Likewise, over time after the COVID-19 pandemic, some studies point to a higher prevalence of depersonalization and low personal fulfillment (43). Other authors still report high levels of emotional exhaustion 4 years after the onset of the pandemic (44). However, there is a lack of longitudinal studies on burnout carried out on health professionals, making it impossible to contrast the evolution of burnout within a single study, and the present study is particularly relevant because of its longitudinal nature. These findings highlight the complexity of burnout syndrome, as its different components have shown different evolutionary trajectories.

Our study assesses the influence that different sociodemographic and occupational variables have on the different components of burnout. With regard to age, it is the younger professionals who report greater emotional exhaustion and depersonalization during the 6 months after the onset of the COVID-19 pandemic. This is in line with what has been stated by other authors, who, during the COVID-19 pandemic, have assessed younger professionals as a group at risk of suffering greater psycho-emotional disturbance (1). As evidenced by our results, female gender and nursing category are also risk factors for a high prevalence of emotional exhaustion at the onset of the pandemic (45, 46). A novel finding of our study is to point out as a risk group for high emotional exhaustion and depersonalization those professionals who performed their work in Primary Care, both in the medium and long term. These findings contrast with the existing literature, which states that those professionals with a higher risk of suffering psychoemotional alteration are those who work in highly complex areas such as emergencies or CCU, or those who work on the front line with infectious-contagious patients (47, 48). This association found in our study could be justified by the massive transfer of health personnel working in primary care to specific centers developed to care for all those COVID-19 patients who could not be offered hospital care due to lack of human and logistic resources, causing work overload and added stress for HCW in primary care.

Emotional exhaustion, along with self-fulfillment, was the component in our study that showed the greatest reduction during the period studied. Emotional exhaustion has undoubtedly been one of the most studied dimensions of burnout, as it is initially the most affected component (49–51), as our findings show. This exponential increase in emotional exhaustion at the onset of the COVID-19 pandemic in HCW has been attributed to increased workload, lack of resources, fear of contagion, and generalized uncertainty (49–51). The longitudinal nature of this research allows us to provide interesting information on the evolution of the dimensions. Our data seem to indicate that as the pandemic progressed and health systems began to adapt better to the situation, emotional exhaustion decreased significantly. According to the existing literature, emotional exhaustion is closely related to levels of depressive symptomatology, especially in the early stages of the pandemic (49, 52). In our study, levels of depression assessed at the onset of the pandemic correlated significantly with emotional exhaustion 6 months after their beginning, reinforcing the importance of mental health at the onset of the crisis. This finding is consistent with previous research highlighting how the emotional impact of pandemic onset was critical for the emergence of depressive symptoms in HCW (53).

With regard to hypotheses 3 and 4, concerning risk and protective variables, respectively, our results support the relevance of some but not all variables. Our findings support hypothesis 5, which proposed a differential role of the mentioned variables in the components of burnout syndrome, hypothesizing a greater effect on emotional exhaustion with respect to the rest of the dimensions.

Thus, with particular regard to the previous hypotheses, depression, cognitive fusion and resilience show a key role, within our study, for the evolution of emotional exhaustion. Those participants who showed high scores for depression and cognitive fusion and low scores for resilience presented a worse evolution for emotional exhaustion. This is consistent with previous studies that relate cognitive fusion (54) and depression (49, 52) to emotional exhaustion, and low levels of resilience with burnout (55, 56).

In contrast to emotional exhaustion, within our study, depersonalization showed an increase during the data analysis period, which again corroborates hypothesis 2, which states the different evolution of each of the dimensions of burnout. This finding is of concern, as depersonalization implies emotional disconnection and dehumanizing treatment of patients, which may have serious implications for the quality of health care (57). Once again, our study shows that primary care professionals are those who report the highest degree of depersonalization during the second time period, although these data are not sustained over time. Previous studies have documented increases in depersonalization in situations of chronic stress, such as the COVID-19 pandemic (58), but our study suggests that this dimension of burnout may be more related to long-term cognitive-emotional factors, such as hopelessness. Hopelessness had already been associated in the existing literature with burnout, however, the literature tends to focus again on total burnout with all its dimensions as a whole or on emotional exhaustion (59). During the pandemic, constant exposure to critically ill patients, scarcity of resources, and accumulated fatigue may have contributed to the development of feelings of hopelessness (15). The chronification of depersonalization, as suggested by the longitudinal nature of our study, implies that HCW are not regaining their ability to reconnect emotionally with their patients, even after emotional exhaustion levels have decreased. The dimension of self-fulfillment shows a markedly significant decrease in our study. Cyclical models of burnout (57) propose the decline in self-fulfillment as the final stage of the syndrome, following emotional exhaustion and depersonalization, conclude in a feeling of incompetence and lack of achievement at work, which negatively affects self-esteem and personal satisfaction (60). The evolution of self-fulfillment in our study is significantly associated with optimism and hopelessness, suggesting that HCW who lack a positive view of the future may feel less competent and less satisfied with their job accomplishments. The role of optimism in burnout prevention has been extensively studied (61). Existing literature documents that optimistic individuals tend to cope with stress in a more adaptive way, finding solutions to problems (62). Our results confirm this association, as professionals who reported higher levels of optimism showed a better evolution in terms of personal fulfillment. On the contrary, those experiencing reduced optimism and higher levels of hopelessness have more difficulties in maintaining their self-fulfillment.

### 4.1 Limitations

The current study has several limitations that should be acknowledged. The sampling method was a non-probabilistic convenience sample, which focused on specific communities and thus lacks generalizability to the broader population. Other limitations relate to the use of self-reports as an assessment method, considering the limitations involved. On the other hand, it is worth noting the possible bias due to sample drop-out. Future lines of research could help to address some of these limitations, especially with regard to the use of self-reports. Although these are essential for the assessment of psychosocial variables, additional behavioral measures associated with burnout, such as absenteeism, sick leave and career drop-out, could be incorporated. Additionally, this study has considered some of the possible psychosocial variables that may be involved. Undoubtedly, there are other additional variables to consider such as positive or negative affectivity, tolerance to ambiguity or emotional regulation, among others. Future research should consider some of the above mentioned psychosocial variables, and their influence on burnout and its evolution in HCW.

### 4.2 Practical implications

The results of this study have important implications for burnout interventions in health care, especially in the context of health policy and practice. On the one hand, the results suggest the need for specific interventions, by considering each of the components of the syndrome. Although further research is needed, emotional exhaustion is shown to be one of the dimensions affected in the short term and intervention programs aimed at reducing depression at times of acute stress (onset of the COVID-19 pandemic), including thought management, seem fundamental. Training programmes that foster resilience and the ability to manage intrusive thoughts (cognitive fusion) could be implemented to address this dimension of burnout (63, 64). Depersonalization and decreased self-fulfillment do not seem to respond to the same pattern and indicate the need for additional interventions. From the cyclical models of burnout (57), it could be hypothesized that both are shown as results of a chronification of a poor management of emotional exhaustion, and in the case of appearing, given the variables associated with its evolution (optimism and hopelessness) therapies more focused on the meaning of existence, such as Acceptance and Commitment Therapy, could be useful. Strategies that promote optimism and reduce hopelessness, such as cognitive and emotional skills training programmes, may be beneficial (65). Interventions aimed at increasing optimism, such as cognitive-behavioral therapies and interventions based on positive psychology, could be useful in improving self-fulfillment among HCW, especially in times of prolonged stress (66, 67). Moreover, the fact that hopelessness plays a crucial role in the decline of self-fulfillment reinforces the idea that addressing these cognitive-emotional factors may be of interest to prevent the chronification of burnout.

On the other hand, it is crucial that health policies include ongoing psychological support measures for health workers, especially in times of crisis such as the COVID-194 pandemic (68). These policies could include the creation of peer support networks and the implementation of organizational wellbeing programmes that address workload and work-life balance (69). Peer support networks allow health professionals to share experiences and strategies for managing stress and burnout. For example, some hospitals have implemented mentoring programmes where more experienced employees support new employees, helping them to adapt to the work environment and manage stress (68). The organizational wellbeing programmes are designed to support the mental and physical health of employees. They may include access to counseling services, physical exercise programmes, and workshops on stress management and resilience (63). An example is the Mayo Clinic’s wellness programme or the Cleveland Clinic’s “Be Well” Program, which offers a variety of resources to support employee wellness, including psychological counseling and fitness programmes (63). On the other hand, work-life balance policies seek to ensure that employees have a healthy work-life balance. They may include flexible work options, family leave, and childcare support (68). Examples of such initiatives include the Mayo Clinic’s “My Time” Initiative or the Massachusetts General Hospital’s “Recharge Days (70).

Similarly, the establishment of patient care policies is a preventive measure to be considered. These policies establish standards for the daily care of patients, including specific procedures for managing medical situations, such as exposure to body fluids or medical emergencies (71). As an example, in the case of the COVID-19 pandemic, a hospital may have a policy detailing how to act in the event of an infectious disease outbreak, ensuring that all employees follow specific protocols to minimize the risk of transmission. These aspects are of particular relevance in the context at hand, given that fear of contagion was a precipitating factor for emotional symptomatology in HCW at the onset of the pandemic (72).

In summary, a multifaceted approach that considers the different dimensions of burnout and associated psychosocial factors is essential to improve the mental health of healthcare professionals and their ability to cope with future crises.

## 5 Conclusion

The findings of our study are relevant for several reasons. Firstly, the longitudinal nature of the study entails an analysis of the long-term evolution of burnout, providing an approach to the current state of health care professionals. On the other hand, the three-dimensional approach to burnout allows us to study the evolution of each variable separately, noting that while emotional exhaustion decreases throughout the study, depersonalization increases and personal accomplishment decreases, which underlines the need for specific intervention approaches for each component. These findings reinforce the importance of psychosocial variables, both short- and long-term, in the evolution of burnout, and suggest that interventions should be tailored to the temporal and emotional context of professionals.

## Data Availability

The original contributions presented in the study are included in the article/supplementary material, further inquiries can be directed to the corresponding author.
